# Monitoring saliva compositions for non-invasive detection of diabetes using a colorimetric-based multiple sensor

**DOI:** 10.1038/s41598-023-43262-z

**Published:** 2023-09-27

**Authors:** Mohammad Mahdi Bordbar, Mahboobeh Sadat Hosseini, Azarmidokht Sheini, Elham Safaei, Raheleh Halabian, Seyed Mosayeb Daryanavard, Hosein Samadinia, Hasan Bagheri

**Affiliations:** 1 Chemical Injuries Research Center, Systems Biology and Poisonings Institute, Baqiyatallah University of Medical Sciences, Tehran, Iran; 2 Health Research Center, Lifestyle Institute, Baqiyatallah University of Medical Sciences, Tehran, Iran; 3 Department of Mechanical Engineering, Shohadaye Hoveizeh Campus of Technology, Shahid Chamran University of Ahvaz, Dashte Azadegan, Khuzestan Iran; 4 Department of Chemistry, College of Sciences, Shiraz University, Shiraz, Iran; 5 Applied Microbiology Research Center, Systems Biology and Poising Institute, Baqiyatallah University of Medical Sciences, Tehran, Iran; 6 Department of Chemistry, Faculty of Science, University of Hormozgan, Bandar-Abbas, Iran; 7 Research Center for Health Management in Mass Gathering, Red Crescent Society of the Islamic Republic of Iran, Tehran, Iran

**Keywords:** Sensors, Medical and clinical diagnostics

## Abstract

The increasing population of diabetic patients, especially in developing countries, has posed a serious risk to the health sector, so that the lack of timely diagnosis and treatment process of diabetes can lead to threatening complications for the human lifestyle. Here, a multiple sensor was fabricated on a paper substrate for rapid detection and controlling the progress of the diabetes disease. The proposed sensor utilized the sensing ability of porphyrazines, pH-sensitive dyes and silver nanoparticles in order to detect the differences in saliva composition of diabetic and non-diabetic patients. A unique color map (sensor response) was obtained for each studied group, which can be monitored by a scanner. Moreover, a good correlation was observed between the colorimetric response resulting from the analysis of salivary composition and the fasting blood glucose (FBG) value measured by standard laboratory instruments. It was also possible to classify participants into two groups, including patients caused by diabetes and those were non-diabetic persons with a total accuracy of 88.9%. Statistical evaluations show that the multiple sensor can be employed as an effective and non-invasive device for continuous monitoring of diabetes, substantially in the elderly.

## Introduction

The dysfunction of insulin hormone results in the failure to transport and store glucose in the cells, making them remain in the bloodstream^1^. This metabolic disorder is known as diabetes, and can occur in children, adolescents, and especially in people over 45 years of age^2^. People with a family history of diabetes, or with poor nutrition and high BMI are more likely to suffer from this disease^3^. Inactivity can promote the development of diabetes. The lack of control over the development of diabetes or its influential factors may cause the patient to suffer from cardiovascular, kidney, limb amputation, and retinopathy damages^4^.

Diabetes is usually controlled by regulating the patient's blood sugar (glucose) through diet changes, insulin injections, and exercise^5,6^. In clinical laboratories, glucose monitoring is performed with common tests such as fasting blood glucose (FBG), oral glucose tolerance (OGTT), random blood glucose test, and hemoglobin A1c test (HbA1c test)^7^. However, the blood sugar of diabetic patients (even those who are required to receive around-the-clock insulin regimens) should be checked at multiple and short intervals throughout the day^8,9^. For this purpose, the use of a glucometer to measure the blood glucose level by the patient has been proposed in recent years^8^. Although this method is highly popular, the patient suffers from the capillary blood sampling process and cost of necessary equipment such as test strips and electronic readers^10^. Probably, an easier way of continuously determining interstitial glucose levels is to use continuous glucose monitoring (CGM) devices such as FreeStyle® Libre 2 or Dexcom G6®^11^. These devices utilize a transmitter to send the information to a receiver or smartphone, allowing for the estimation of the blood glucose level by the relevant software^11^. Nevertheless, the performance of the CGM devices is limited by their high price. Also, some factors such as a 10–20% difference between interstitial glucose level and blood glucose, lack of correct calibration, and drug interventions can have negative effects on the detection accuracy^12^.

Many efforts have been made to develop non-invasive methods for determination of glucose in body fluids (e.g., saliva)^13^. In these methods, the sample is collected by the person running the experiment without any skills, and the possibility of contamination is low^14^. Furthermore, reported data for the non-invasive methods have shown a good correlation between glucose concentrations in saliva and blood^15^. However, the assay devices must have high sensitivity because the glucose concentration in the saliva of diabetic patients is several tens of times lower than that in the blood^16^.

A suitable alternative is to assess the level of total compounds in the saliva sample of diabetic patients using high-performance gas and liquid chromatography or mass and infrared spectrometry^17^. It should be noted that the concentration of metabolites and proteins in the saliva of a patient may increase or decrease when compared to that of a healthy person. Based on previously published studies, the concentration of compounds such as alanine, pyroglutamine, oxoproline, citrulline, ornithine, leucine, alpha-hydroxyisovalerate, 2-hydroxybutyrate, alpha-ketobutyrate, 4-hydroxyphenylpyruvate, glucose, 1,3-dihydroxyacetone, lactate, xylonate, fructose, gluconate, acetoacetate, heptanoate, ethanolamine, benzoate and metformin is different in saliva samples of healthy and diabetic individuals^17–20^. Having high accuracy and sensitivity are among the prominent features of instruments used for evaluating the metabolic profile, although their efficiency is limited due to the cost, laboratory conditions for analysis, and the need of a skilled operator^21^.

Changes in the constituents of complex samples can be observed with multiple sensors. Basically, these devices consist of sensing elements with different reactive properties that interact with chemical compounds with diverse functional groups, while also producing electrochemical, thermal, mass or colorimetric signals^22^. In this way, responses caused by color changes are easily observable by the user.

The feasibility of these sensors increases when they are immobilized on a paper substrate, arising from their good flexibility^23^. As well, they can be fabricated in desired dimensions, transferred to the analysis environment, and easily removed after use with producing less contamination. On the other hand, the selection of sensing elements from various materials such as dyes, nanoparticles (NPs) with different coating agents, and tetrapyrrole macrocycles can have a positive effect on the sensing performance, sensitivity, and selectivity of the sensor^24^. Paper-based multiple sensors have been used to detect COVID-19^25^, cancer^26^, urinary tract infection^27^ and sepsis^28^, and to measure pesticides^29^ and mycotoxins^30^, or evaluate the quality of fuels^31^ and foods^32,33^.

This study aims to develop a multiple sensor based on paper substrates with an origami structure in order to monitor metabolic changes caused by diabetes. The paper substrate consists of two layers folded over each other. The saliva sample is injected into one layer and penetrates vertically into the paper tissue to react with the sensor components located in the second layer. With the help of this structure, it is possible to reduce the interference caused by the viscosity of saliva, while also decreasing the analysis time and preventing the movement of sensing elements to the sides of the detection zones^34,35^. In this sensor, (with cobat, zinc and copper central cores), organic dyes (mixed with reagents such as tetrabutylammonium hydroxide and phenylboronic acid), and silver NPs (containing thiomalic acid, L-arginine, and chitosan coating agents) play the role of receptors. Depending on their structural properties, the receptors trap the compounds in the saliva sample, followed by changes in their color. More precisely, this study attempts to present a special color pattern for diabetic patients, being different from the response obtained for healthy individuals and thus allowing for monitoring the severity of diabetes progression. Also, the intensity of the color changes should have a good correlation with the blood glucose level.

## Results and discussion

Despite the methods utilizing blood samples to determine the severity of diabetes, investigation of the development of this disease by analyzing saliva secretions would be an easier approach. In this study, a sensor was designed to monitor changes in the level of salivary metabolites associated with diabetes. To achieve this goal, it was first checked whether the sensor could detect the difference in the type or concentration of saliva compositions of diabetic and non-diabetic individuals. In the next step, the correlation of the changes in the color intensity of all sensor components (or a certain sensing receptor) with the severity of the disease, blood glucose level, individual's mobility, and other factors was investigated. The answers to the above questions and the analysis of the data collected in this study are presented in the following sections.

### Optimization of parameters

Optimization is done to achieve the best response of the designed sensor. The output of the sensor should separate diabetic patients from non-diabetic patients with high sensitivity and specificity and have a reasonable correlation with the severity of the disease. For this purpose, the optimization process was carried out based on the discrimination ability function (DAF), calculating the ratio of inter-group variance to intra-group variance for each parameter^21^. The optimum conditions were obtained when DAF had reached the highest numerical value^21^. The parameters such as the concentration of sensing receptors, the mixing ratio of reagents and organic dyes, and the interaction time could play effective roles in the sensor responses.

**Figure 1:**
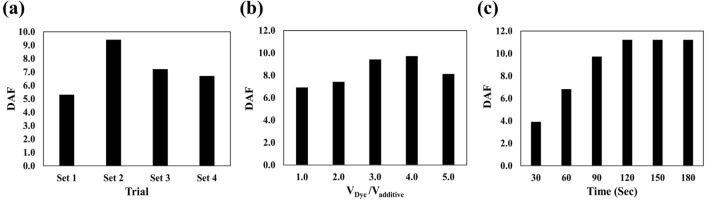
The optimization results for (**a**) sensing receptors concentration, (**b**) the volume ratio of organic dye and additive in a mixture and (**c**) the time of interaction between sensor and salivary metabolites. In Fig. 1a, each set was introduced as following: set 1: (porphyrazines: 1.0 mg mL^−1^, organic dyes: 2.0 mg mL^−1^, and AgNPs: 1.0 mg mL^−1^), set 2: (porphyrazines: 1.5 mg mL^−1^, organic dyes: 2.0 mg mL^−1^, and AgNPs: 1.0 mg mL^−1^), set 3: (porphyrazines: 2.0 mg mL^−1^, organic dyes: 2.0 mg mL^−1^, and AgNPs: 1.0 mg mL^−1^) and set 4 (porphyrazines: 2.5 mg mL^−1^, organic dyes: 2.0 mg mL^−1^, and AgNPs: 1.0 mg mL^−1^).

The sensing receptors were prepared in four different concentration sets: porphyrazines (1.0, 1.5, 2.0, and 2.5 mg mL^−1^), organic dyes (1.0, 2.0, 3.0, and 4.0 mg mL^−1^), and AgNPs (0.5, 1.0, 1.5, and 2.0 mg mL^−1^). Note that in this step, the reagents were combined with the organic dyes in a ratio of 1:1. Based on the data presented in Fig. 1a, performance of the sensor is maximized when all sensing receptors are fabricated by the second set of concentrations: (porphyrazines: 1.5 mg mL^−1^, organic dyes: 2.0 mg mL^−1^, and AgNPs: 1.0 mg mL^−1^). The receptors prepared by lower concentrations have fewer interaction sites, thus lacking the ability to react with sufficient amounts of metabolites. Also, in the higher concentrations, clear changes in the color of the receptors are not visually and digitally received due to the high intensity of their initial color^36^.

The organic dyes were mixed with PBA and TBOH reagents in the sensor structure, and the ratio of the mixture components could affect the sensor responses. Therefore, five solutions of each organic dye mixed with its corresponding reagent were prepared, so that the volume ratio of the organic dye to the reagent changed from 1:1 to 5:1. Figure 1b shows that the performance of the sensor is directly related to the increase in the dye concentration. Probably in the ratio of 1:1, the active sites in organic dyes are covered by the reagent and cannot interact with the reaction product of the reagent and the analyte. The maximum responses were achieved in the molar ratio of 4:1 (organic dye:reagent). In continuance, the concentration of the reagent in the reaction medium is too low, which cannot react with the analyte and produce enough product to interact with the organic dye, so that, the DAF value decreases^36^.

The interaction between the saliva sample compounds and the sensing elements must be performed in an appropriate period of time to make the reaction reach equilibrium. By the progress of the reaction in a certain period of time, the sensor's response gradually increased. After 4 min, the color changes was fixed, which means that the reaction reached equilibrium and the texture of the sensing elements was saturated with analyte (Fig. 1c). This time was selected as the sensor response time for data collection.

### Evaluation of sensor responses

**Figure 2:**
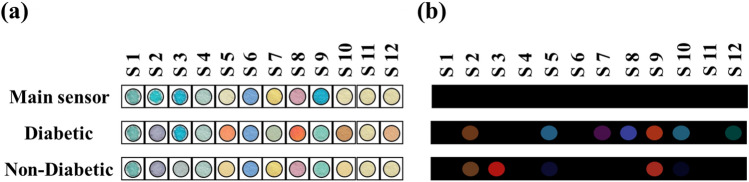
The analysis results: (**a**) color responses of sensor after exposing to salivary metabolites and (**b**) the color maps extracting from image analysis. The sensor was fabricated based on optimized conditions and its response was captured after 2 min.

Since the multiple sensor must be able to detect a wide range of salivary species at low concentrations, it is necessary for its components to include sensing receptors with diverse interaction sites. The receptors can participate in Lewis acid–base (tetrapyrrole macrocycles) or Brønsted acid–base (organic dyes mixed with PBA or TBOH) interactions. Of course, AgNPs can have electrostatic, H-bonding, or covalent interactions with salivary metabolites due to the inherent characteristics of the central metal and the structural properties of the coating agents. According to Fig. 2, only two tetrapyrrole macrocycles i.e. Zn-Pa (S2) and Cu-Pa (S3), respond to the chemical compounds of saliva. The former interacts with the common metabolites of the saliva samples of diabetic and non-diabetic groups, and the latter responds to compounds found in the salivary secretions of a non-diabetic individual. Among the organic dyes mixed with TBOH, only MR (S5) is capable of identifying the salivary species. The color intensity of this indicator is varied in the pattern obtained for diabetic and non-diabetic groups, indicating that the resulting sensor can track the changes in the concentration of metabolites. Unlike the previous set of sensing receptors, the color of organic dyes combined with PBA (including BP (S7) and BR (S8)) changes in the presence of salivary metabolites of the diabetic group. However, MB + PBA (S9) can react with chemical species of both studied groups. As shown in Fig. 2, AgNPs synthesized with CS (S12) accumulate only in the presence of diabetic saliva samples, and their color changes from yellow to brown. This event is observed for TMA-AgNPs (S10) after exposing them to the saliva secretions of diabetic and non-diabetic volunteers.

While the variations in the saliva compositions of participants can be detected by TMA-AgNPs, the responses of Co-Pa, TB and BB integrated with TBOH and Arg-AgNPs are not received. It should be noted that these receptors are discolored when the specific markers of other diseases such as lung, kidney, gastrointestinal and COVID-19 diseases as well as being a smoker exist in the saliva secretion of a diabetic or non-diabetic individual. For all volunteers, the results of sensor were presented in Figs. S1 and S2.

### Discrimination results

The feasibility of the multiple sensor in the detection of a difference between the metabolic profiles of diabetic and non-diabetic individuals was investigated using PCA-LDA. In this regard, a matrix containing 90 data vectors of 36 members was prepared, and then given to the pattern recognition algorithm. Based on the statistical information summarized in Table 1, the sensor is found to be able to classify 41 diabetic and 39 non-diabetic individuals in their respective group. Most of the members of the first group have an FBG level > 100 mg/dL, whereas the FBG level of the participants in the second group is less than 100 mg/dL. Nevertheless, some individuals who are misclassified have FBG and HGA1c values in the range from 100 to 106 and 5.9 to 6.5, respectively.

**Table 1:** Discrimination parameters.

Error rate: 11.1% Accuracy: 88.9%		
Sample	Sensitivity (%)	Specificity (%)
Diabetic	91.1	86.6
Non-diabetic	86.6	91.1

**Figure 3:**
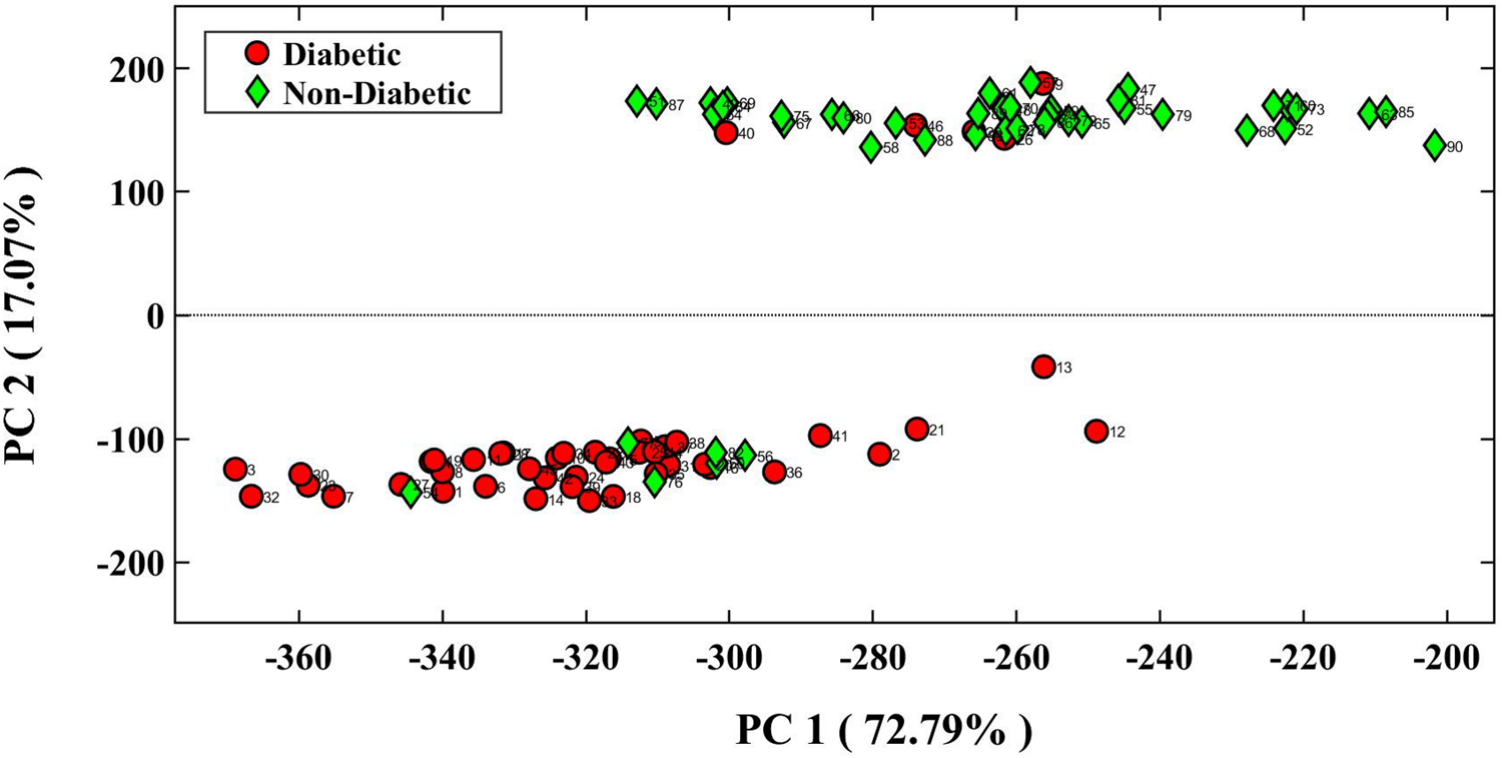
The score plot obtained from PCA-LDA analysis for discrimination of diabetic and Non-diabetic participants. The sensor was fabricated based on optimized conditions and its response was captured after 2 min.

The data distribution in the space of the first two principal components (having 89.86% of the total extracted variance) is shown in Fig. 3. As can be inferred, by limiting the data of the PC2 axis in the range of 95–205, while also moving from − 200 to − 320 on the PC1 axis, the blood glucose levels of the non-diabetic group are correctly recognized in the range of 79–105. On the other hand, by limiting the PC2 axis from − 50 to − 200 and moving from − 240 to − 380 on the PC1 axis, the blood glucose levels of diabetic participants change from 89 to 304. Therefore, a good linear correlation exists between the FBG values and the magnitude of the PC1 axis, thereby allowing for the estimation of the blood glucose level of an individual after receiving the information from the PC1 data.

Figure 3 indicates that the FBG values for the diabetic participants classified in the range of − 240 to − 290 on the PC1 were low, whereas the corresponding HGA1c values were above 7.0. However, these participants were active for more than 8 h, whereas the patients classified in the range of − 340 to − 380 performed less than 1 h of activity or had no activity. In other words, the high mobility improves the body's reaction to insulin and regulates the FBG level^37^.

The discrimination between the two studied groups was performed with the help of the total response of the sensor. By calculating the Euclidean norm of the data vector provided by the image analysis, a certain numerical value was obtained for each sample. The average of Euclidean norms related to the analysis of 90 saliva samples, diabetic and non-diabetic groups were calculated to be 343.39 (± 33.26), 358.31 (± 27.98) and 328.47 (± 31.60), respectively. Accordingly, the difference between the means of the studied groups was numerically equal to (29.84), being statistically significant based on the results of the two independent sample t-tests (*P*-value < 0.001). Therefore, with a confidence level of 95%, the data greater than the total mean value (343.39) belong to the diabetic group, and the values smaller than it are classified into the non-diabetic group.

### Correlation between sensor response and blood glucose level

The correlation between color changes of each sensing receptor and blood glucose level (obtained from the clinical laboratory analyses) was investigated, enabling us to find a model to estimate blood glucose levels using the analysis of saliva sample metabolites. By categorizing the blood glucose level of the participants into the different intervals presented in Fig. S3, it was found that the discoloration of (Methyl red + TBOH) has a correlation with the specified categories. The Pearson coefficient of this correlation was calculated to be 0.726 (*P*-value < 0.001). On the other hands, a new correlation was observed between the TMA-AgNPs and blood glucose level, having a Pearson coefficient of 0.871 (*P*-value < 0.001). The model proposed for both receptors can provide a good estimate of blood glucose levels by analyzing the individual’s saliva sample. Compared to the sensor made of organic dye, the sensing receptors prepared based on NPs show higher performance in determination of the blood glucose level with smaller intervals due to their unique characteristics and sensitivity.

### Effect of age on the sensor response

Can the observed metabolic changes be affected by the age of participants? To answer this question, the total response of the sensor was calculated for each sample, and its correlation with the age of the volunteers was investigated. According to the statistical data, Pearson coefficients are equal to 0.166 and 0.252 for the diabetic and non-diabetic groups, respectively. Moreover, the respective *P*-values of the groups are calculated to be 0.271 and 0.098. Since an inappropriate relationship exists between the two variables, it can be concluded that the age changes do not affect the response of the proposed sensor.

### Reproducibility and stability

The reproducibility of the sensor response was studied by fabricating five sensors based on the origami structure. In this case, 40.0 μL of the saliva sample of diabetic and non-diabetic individuals was injected into the injection part of the sensor. The Euclidean norm was calculated for each analysis, followed by estimating the relative standard error (RSD %) of five determinations for a certain sample. The data presented in Fig. S4 show that, using the designed sensor, the diabetes diagnosis through the analysis of the salivary metabolites was highly reproducible as the RSD values of diabetic and non-diabetic samples are 6.03% and 6.71%, respectively.

To evaluate the stability of the array based sensor, the total response obtained as Euclidean norm, was investigated for a period of time. In this experiment, the sensor was not exposed to saliva sample but it was kept in the laboratory environment at 25°. The trends in the sensor color changes were reported in Fig. S5. As illustrated, the device lasted up to 25 days against the physical and the chemical changes in the environment. After that, a gradual color change of receptors was observed.

Table 2 shows the efficiency of some fabricated sensors for detection of diabetes through the analysis of salivary metabolites. These sensors either have electrochemical transducers or follow a colorimetric mechanism. In these assays, only saliva glucose is detected through an enzymatic process or by molecular imprinted polymer (MIP) and nanozyme^34,38–42^. Although, these methods can detect the trace amounts of glucose in a standard solution, the concentration of this marker in the saliva sample is ten times lower than the blood sample, also, the presence of this analyte in salivary secretions can be caused by non-diabetic metabolic processes. Therefore, the reliability of the data obtained from glucose-sensitive sensors in the saliva sample can be associated with limitations. Compared to these studies, pattern-based methods tracked all the compounds in the saliva sample and created a unique diagnostic pattern for diabetic and non-diabetic subjects, which can be reliable data. Compared to the previous methods, detection of the metabolites has been done with a sensing tool including inexpensive non-enzymatic sensors, which is portable and easily manufactured, can distinguish diabetic people from non-diabetic people with a high degree of accuracy, and its response has a good linear correlation with the blood glucose level obtained from clinical methods.

**Table 2:** Comparison between the performances of the developed sensors for the diagnosis of diabetes using saliva samples.

Method	Species	Sensing element	Sensitivity	Selectivity	Volume of sample	Response time	Ref
Colorimetric	Glucose	DEPDA\4CN\HRP	1.0 mg/dL	–	0.3 µL/min	4 min	38
Electrochemical	Glucose	MIP\Au-SPE	0.59 μg/mL	100%	–	30 min	39
Colorimetric	Glucose	GOx\HRP\TBHBA	0.37 mg/dL	100%	2.0 µL	–	40
Electrochemical	Glucose	NiCl(OH) nanosheet array	0.29 μM	100%	–	< 5 s	41
Electrochemical	Glucose	CuO/rGO/CNF/GCE	0.1 μM	100%		< 3 s	42
Colorimetric	Glucose	Sericin-AgNPs\TMB	0.37 mg/dL	100%	10.0 μL	7 min	34
Colorimetric	All saliva metabolites	Array of PO, OD and AgNPs	91.1%	86.6%	40.0 μL	4 min	This work

*DEPDA* N,N′ -Diethyl-p-phenylenediamine, *4CN* 4-Chloro-1-naphthol, *HRP* Horseradish peroxidase, *MIP* Molecular imprinted polymer, *Au-SPE* Screen printed gold electrode, *GOx* Glucose oxidase, *TBHBA* 2,4,6-tribromo-3-hydroxy benzoic acid, *rGO* reduced graphene oxide, *CNFs* Carbon nanofibers, *GCE* Glassy carbon electrode, *PO* Porphyrazines, *OD* Organic dye, *AgNPs* Silver nanoparticles, *TMB* 3,3′,5,5′-tetramethylbenzidine.

## Conclusion

This study has used a user-friendly method based on the analysis of salivary metabolites in order to facilitate and speed up the process of diabetes diagnosis. The method responses were based on the changes in the color of sensing receptors of the sensor, so that the interpretation of the results did not require an operator. The sensor could detect the difference between the chemical compositions of saliva samples of diabetic and non-diabetic individuals, while also estimating the blood glucose level or range, determining the mobility of each individual, and providing a reproducible response for the analysis of a certain sample. The color of the receptors changed only due to the changes in the metabolic profile of the saliva samples. The multiple sensor was fabricated in such a way that some factors such as the chemical markers (related to other diseases or diet) not only did not act as interferences for diabetes detection, but could also be detected simultaneously with a specific multiple sensor. Simplicity, portability, and having a reliable response can be the advantages of this sensor to replace the current methods. However, since the sample must be centrifuged, this sensor can currently be used in clinical laboratory. In the next design, the sensor structure can be refined by embedingg the multiple layers in the sensor strcture for the filtration and pre-treatment process. Also, by providing an application, the immagging process, analsis the color changes of sensor and reporting the final response can be conducted in the shortest possible time. Adding receptors that can detect the effects of different drugs for the treatment of diabetes is also another purposes that we will address in future studies.

## Method

### Materials

The following compounds were obtained from Sigma Aldrich: thiomalic acid (TMA), L-arginine (Arg), thymol blue (TB), methyl red (MR), bromophenol blue (BB), bromopyrogallol red (BR), methyl blue (MB), phenylboronic acid (PBA), and tetrabutylammonium hydroxide (TBOH). The chemicals including chitosan (CS), bromocresol purple (BP), silver nitrate (AgNO_3_), glacial acetic acid, trisodium citrate, and ethanol (EtOH) were purchased from Merck. Tetrapyrrole macrocycles consisting of tetramethyl quaternized tetracationic porphyrazines (tetramethyl tetra-3,4-pyridinoporphyrazinato cobalt(II) [Co(3,4-tmtppa)]^4+^, tetramethyl tetra-3,4-pyridinoporphyrazinato zinc(II) [Zn(3,4-tmtppa)]^4+^, and tetramethyl tetra-2,3-pyridinoporphyrazinato copper(II) [Cu(2,3-tmtppa)]^4+^) were prepared by the methods published previously^43–45^. The substrate of the sensor was made of Whatman® Grade NO.2 filter paper.

### Instrumentation and software

AutoCAD 2016 was used to create the design of the origami-based sensor. This pattern was plotted on the sensor substrate using HP LaserJet printer 1200. A micropipette (BRAND Transferpette® S, Germany) was employed to inject the sensor components into the detection zones. A CanoScan LiDE 220 scanner was used to capture the sensor photos. The color of sensing elements was investigated by ImageJ (1.51n, National Institutes of Health, USA). Discrimination and statistical analyses were conducted by MATLAB R2015 and SPSS (Version 22; Chicago, IL, USA).

### Preparation of sensor components

The multiple sensor was composed of three Ag nanoparticles (AgNPs) synthesized by TMA, Arg, and CS. The yellow color solution of TMA-AgNPs was created by mixing the aqueous solution of 0.1 molL^−1^ AgNO_3_ (250.0 µL) and 0.1 molL^−1^ trisodium citrate (250.0 µL) in deionized water (100.0 mL), followed by adding 2.5 × 10^–3^ molL^−1^ of NaBH_4_ (6.0 mL). Finally, 1.0 × 10^–2^ molL^−1^ of TMA (500.0 µL) was injected into the solution. The synthesis process was performed on a stirrer during 1 h, and the reaction temperature was adjusted to 25 °C^46^. To prepare Arg-AgNPs, 0.01 W/V% of NaBH_4_ solution was initially added to 1.0 × 10^–4^ molL^−1^ of AgNO_3_ (50.0 mL) drop by drop, and 10.0 mL of the resulting solution was then mixed with 1.0 × 10^–3^ molL^−1^ of Arg solution (1.0 mL). The mixture was centrifuged to remove the unreacted Arg^47^. To obtain CS-AgNPs, the solution of 1.0 × 10^–3^ molL^−1^ AgNO_3_ (2.0 mL) was poured into a flask containing 47.0 mL of CS (0.2 W/V %). The resulting mixture was stirred vigorously at 25 °C. After 30 min, 8 mg mL^−1^ of NaBH_4_ solution was added to the container slowly until the appearance of the yellow color solution of CS-AgNPs^48^.

The three synthesized NPs were powdered and homogenized. Ultimately, a certain amount of the powders was dispersed in deionized water to prepare NP solutions with a specific concentration. To prepare the solution of other sensing receptors, the organic dyes and tetrapyrrole macrocycles were dissolved in EtOH and deionized water, respectively. The prepared solutions of dyes were mixed with TBOH or PBA reagents under an optimized volume ratio.

### Sensor structure

**Figure 4:**
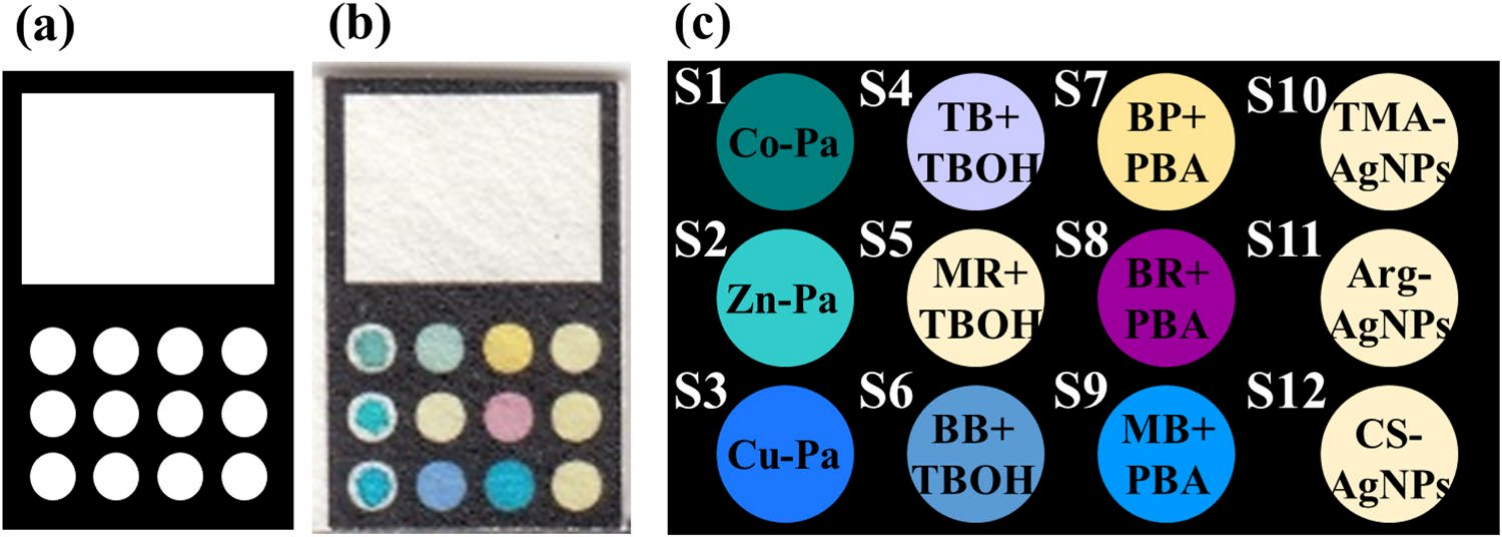
The proposed multiple sensor: (**a**) the designed pattern, (**b**) the fabricated sensor and (**c**) the list of sensing receptors. Co-Pa: Tetramethyl tetra-3,4-pyridinoporphyrazinato cobalt(II) [Co(3,4-tmtppa)]^4+^, Zn-Pa: Tetramethyl tetra-3,4-pyridinoporphyrazinato zinc(II) [Zn(3,4-tmtppa)]^4+^, Cu-Pa: Tetramethyl tetra-2,3-pyridinoporphyrazinato copper(II) [Cu(2,3-tmtppa)]^4+^, TB: Thymol blue , MR: Methyl red, BB: Bromophenol blue, BP: Bromocresol purple, BR: Bromopyrogallol red, MB: Methyl blue, TMA: Thiomalic acid , Arg: L-arginine , CS: Chitosan and TBOH: Tetrabutylammonium hydroxide.

The multiple sensor was implemented on a paper substrate with an origami structure. The design of this sensor (1.5 cm in length and 1.0 cm in width) was performed in the AutoCAD environment. The sensor pattern was printed on a paper substrate, followed by transferring it to an oven to be baked and to form black hydrophobic regions^49^. In this condition, The printer's ink was melt, penetrated into the texture of the paper and fill it's holes. It was resulted in blocking the path of sample flow in this area and creating the hydrophobic region. The origami substrate consisted of the two following parts: one with small circles for placing the sensing receptors (0.2 μl), and the other with a white rectangle for sample injection. Figure 4 shows a view of the prepared sensor and the list of the sensing receptors.

### Participants

The participants were two groups: confirmed diabetic patients, and non-diabetic individuals without any diabetes-related disease. These groups included 51 men and 39 women. The average age of the diabetic and non-diabetic groups was 59.97 (± 8.73) and 53.66 (± 10.34), respectively. Volunteers were selected from among the individuals who were referred to the clinical laboratory of Baqiyatallah Hospital in 2021–2022. They studied the steps and conditions of the reaction, and gave their consent to participate in the experiment. All volunteers were not to consume food or beverage for 8–12 h before the test. The patients were either new samples or people who were taking medication. The disease of these people was considered by evaluating all the effective factors such as fasting blood glucose (FBG), two-hour postprandial glucose (Glucose 2 h.p.p), triglycerides (TG) and Hemoglobin A1C (HG A1C). Table 3 presents the demographic information of the studied population. All experiments were performed in accordance with relevant guidelines and regulations.

**Table 3:** The demographic information of diabetic and Non-diabetic participants.

Variable	Non-diabetic participant	Diabetic patient
Total sample	55	53
Excluded sample	11	7
Studied sample	45	45
Sex		
Male	20	26
Female	25	19
Age (Mean ± SD)	53.66 (± 10.34)	59.97 (± 8.73)
FBG*	96.39 (82–109)	146.37 (84–287)
Glucose 2 h.p.p**	–	225.21 (149–328)
TG***	168.72 (52–625)	184.62 (81–477)
HbA1C****	5.53 (5–6.3)	7.54 (5.6–11.7)

*FBG: Fasting blood glucose.

**Glucose 2 h.p.p: Two-hour postprandial glucose.

***TG: Triglycerides.

****HbA1C: Hemoglobin A1C.

### Sample preparation

The salivary secretions of the participants (2.0 ml) were collected into a sterile falcon. This was conducted 15 min after mouth washing in order to remove physical particles in the saliva sample. The collected saliva was centrifuged at 10,000 rpm and 4 °C for 10 min, thereby separating the remaining impurities from the original sample^25^. Moreover, 40 μL of the purified sample was used for colorimetric analysis.

### Sensing evaluation

An illustration of the multiple sensor performance is shown in Fig. S6. The origami structure of the sensor (Fig. S6a) allows the two parts of the paper substrate to comfortably overlap with each other (Fig. S6b). The folded paper is pressed between the two holders (Fig. S6c), and the analyte is injected into the sensor through the cavity embedded in the upper holder (Fig. S6c). The sample is spread on the substrate surface due to the capillary nature of the paper^50^. The saliva metabolites are bonded with the sensing receptors. The result of this interaction is a change in the color of the sensor components, being easily visible to the naked eye (Fig. S6d). However, the response of the sensor was tracked with a scanner (Fig. S6e), and its color values were numerically extracted using the image analysis software (ImageJ) (Fig. S6e). Three numerical values associated with the three color spaces (red, green, and blue) were recorded for each sensing receptor. Finally, the sensor response was presented as a vector of 36 data points (12 sensing receptors × 3 color spaces). Foe each studied sample, a unique color map was extracted (Fig. S6f).

### Evaluation of sensor performance

The performance of the sensor was evaluated qualitatively (i.e., discrimination between salivary compositions of diabetic and non-diabetic groups) and quantitatively (i.e., calculation of the sensing receptor response and determination of its relationship with the blood glucose level). In this respect, the data matrix containing the response vectors of all the analyzed samples was initially prepared, followed by utilizing it as an input to pattern recognition algorithms (e.g., principal component analysis-linear discriminant analysis (PCA-LDA)) in order to evaluate the sensor discrimination performance. The classification of the studied groups was also performed by comparing the average of sensor total responses for the diabetic group with that obtained for the non-diabetic group using two independent sample t-tests. The total response of the sensor was the Euclidean norm of the response vectors obtained for each sample. The Pearson correlation coefficient was used to investigate the correlation between the sensing receptor response and some parameters such as age and blood sugar level.

### Compliance with ethical standards

The research ethics committee of Baqiyatallah University of Medical Sciences has approved the project (Approval ID: IR.BMSU.REC.1400.100).

### Supplementary Information

Supplementary Figures.

## Data Availability

The datasets used and/or analyzed during the current study are available from the corresponding author on reasonable request.
