# Supplementary material for: Monitoring saliva compositions for non-invasive detection of diabetes using a colorimetric-based multiple sensor
Source: Sci Rep. 2023 Sep 27;13:16174. doi: 10.1038/s41598-023-43262-z (PMC10533566; doi:10.1038/s41598-023-43262-z)
Supplement: Supplementary file 1 — Supplementary Figures. [file 41598_2023_43262_MOESM1_ESM.docx]

Supporting Information

for

**Monitoring saliva compositions for non-invasive detection of diabetes using a colorimetric-based multiple sensor**

*Mohammad Mahdi Bordbar^1^, Mahboobeh Sadat Hosseini^2^, Azarmidokht Sheini^3^, Elham Safaei^4^, Raheleh Halabian^5^, Seyed Mosayeb Daryanavard ^6^, Hosein Samadinia ^1^, Hasan Bagheri^1,7*^*

*^1^ Chemical Injuries Research Center, Systems Biology and Poisonings Institute, Baqiyatallah University of Medical Sciences, Tehran, Iran*

*^2^ Health Research Center, Lifestyle Institute, Baqiyatallah University of Medical Sciences, Tehran, Iran*

*^3^Department of Mechanical Engineering, Shohadaye Hoveizeh Campus of Technology, Shahid Chamran University of Ahvaz, Dashte Azadegan, Khuzestan, Iran*

*^4^ Department of Chemistry, College of Sciences, Shiraz University, Shiraz, Iran*

*^5^ Applied Microbiology Research Center, Systems Biology and Poising Institute, Baqiyatallah University of Medical Sciences, Tehran, Iran*

*^6^Department of Chemistry, Faculty of Science, University of Hormozgan, Bandar-Abbas, Iran*

*^7^ Research Center for Health Management in Mass Gathering, Red Crescent Society of the Islamic Republic of Iran, Tehran, Iran*

Corresponding author: [h.bagheri@bmsu.ac.ir](mailto:h.bagheri@bmsu.ac.ir)


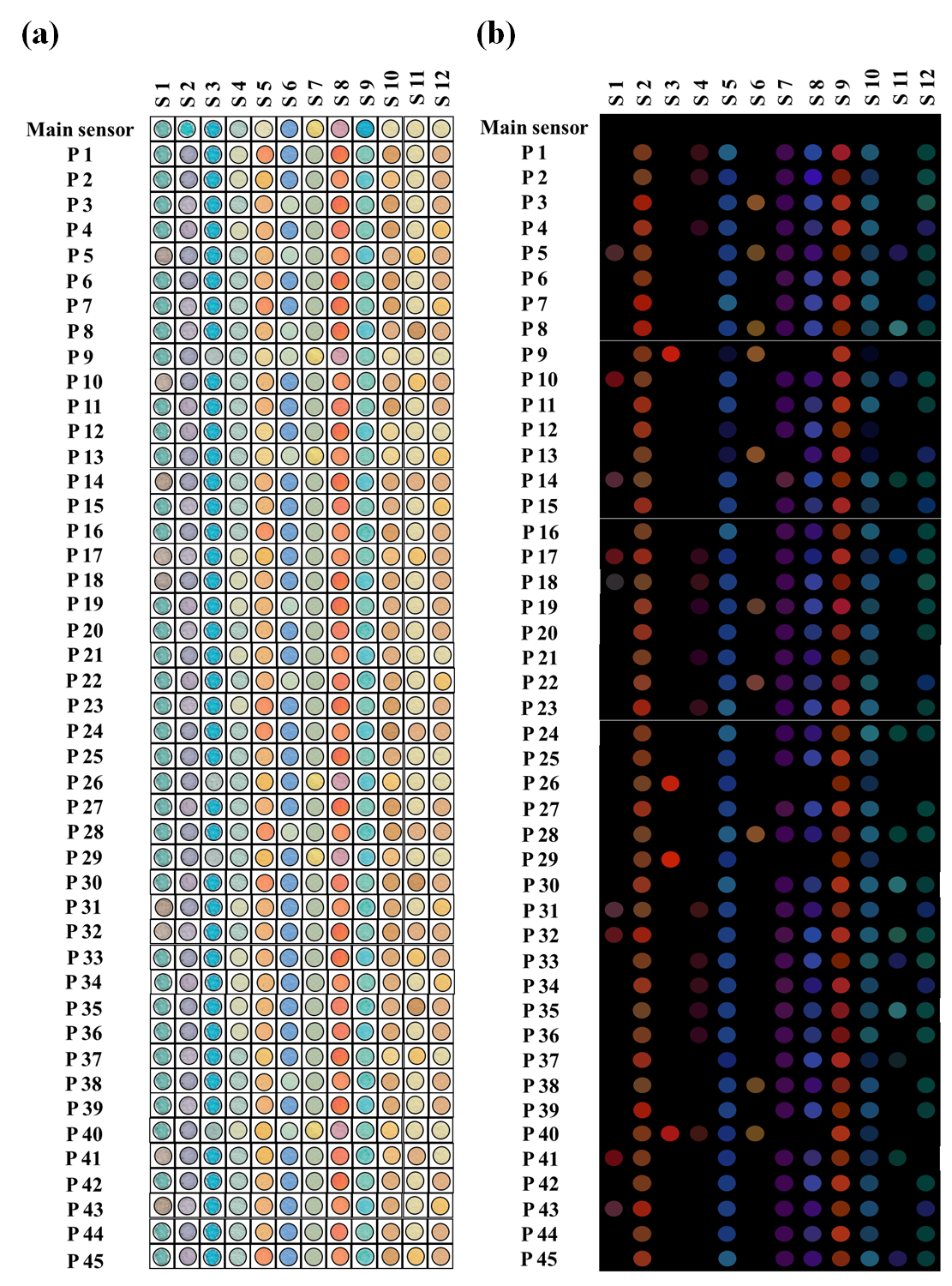


**Fig. S1**. The color responses (a) and the color maps (b) obtained by analyzing the salivary metabolites of diabetic participants which are indicated by (**P**) in this Figure. The sensor was fabricated based on optimized condition and its response was captured after 2 min.


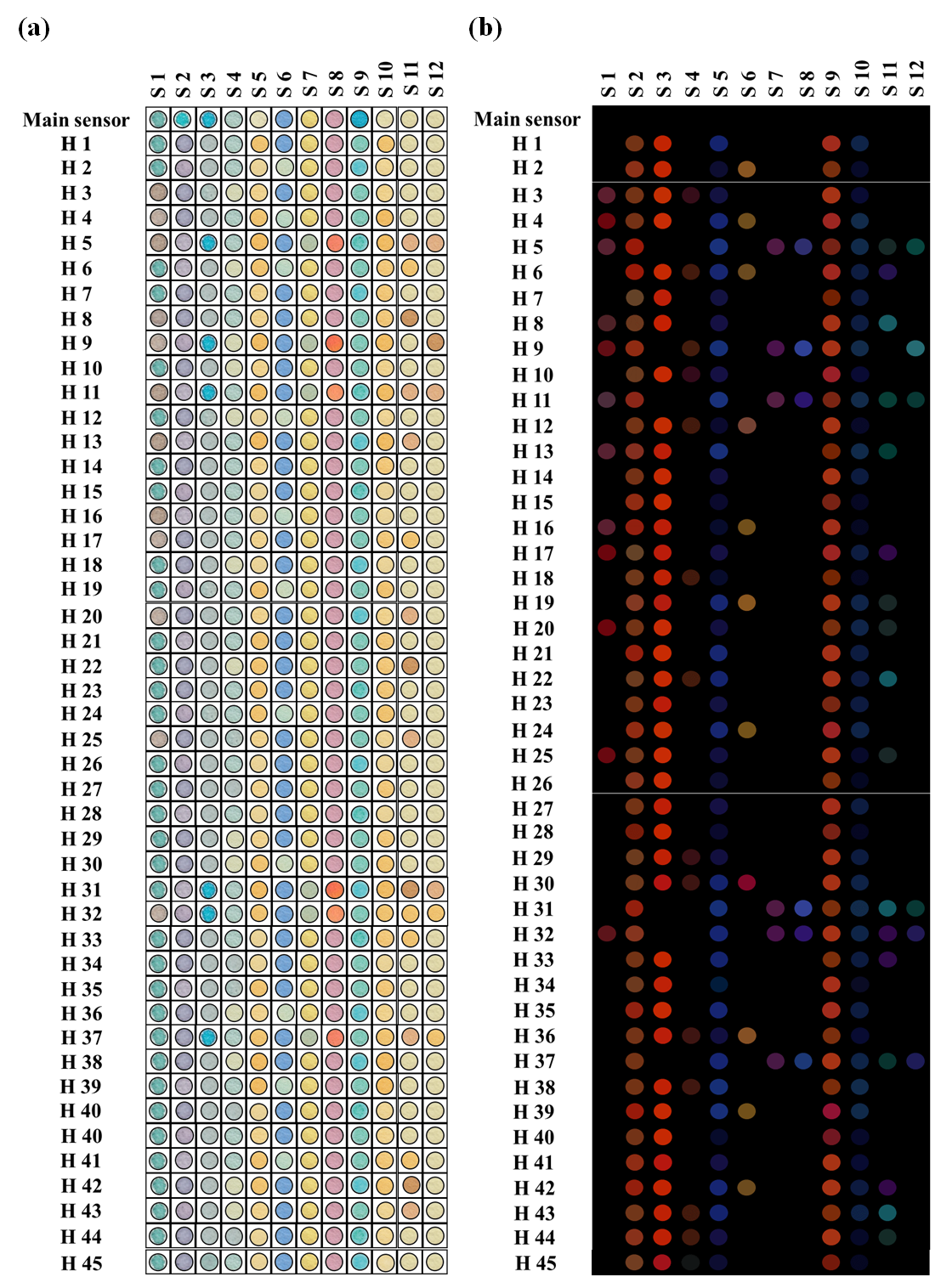


**Fig. S2**. The color responses (a) and the color maps (b) obtained by analyzing the salivary metabolites of Non-diabetic participants which are indicated by (**H**) in this Figure. The sensor was fabricated based on optimized condition and its response was captured after 2 min.


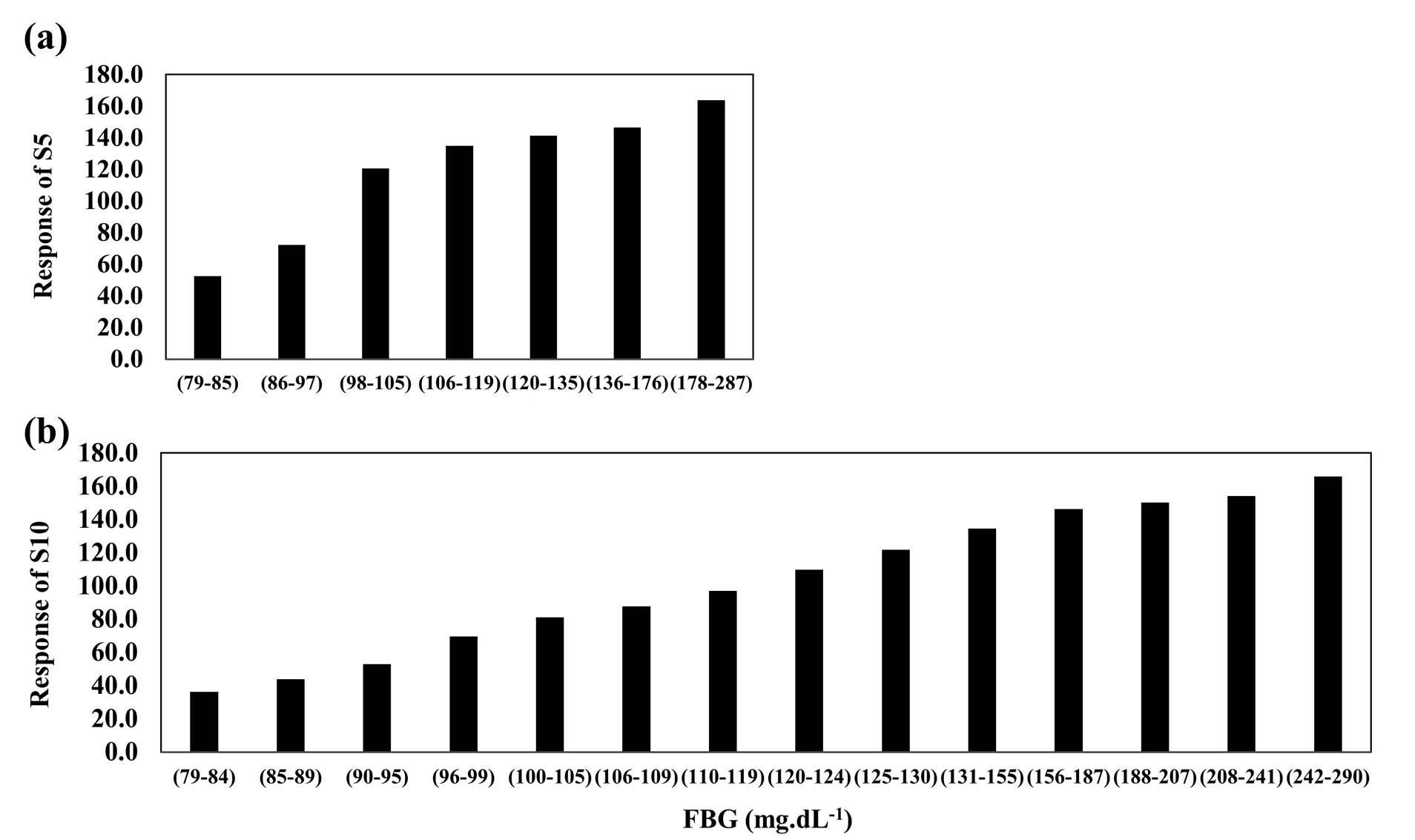


**Fig. S3**. The relationship between the responses of (a) sensing receptor (S 5) and (b) sensing receptor (S 10) with the value of blood glucose obtaining from the clinical laboratory analyses. The sensor was fabricated based on optimized condition and its response was captured after 2 min.


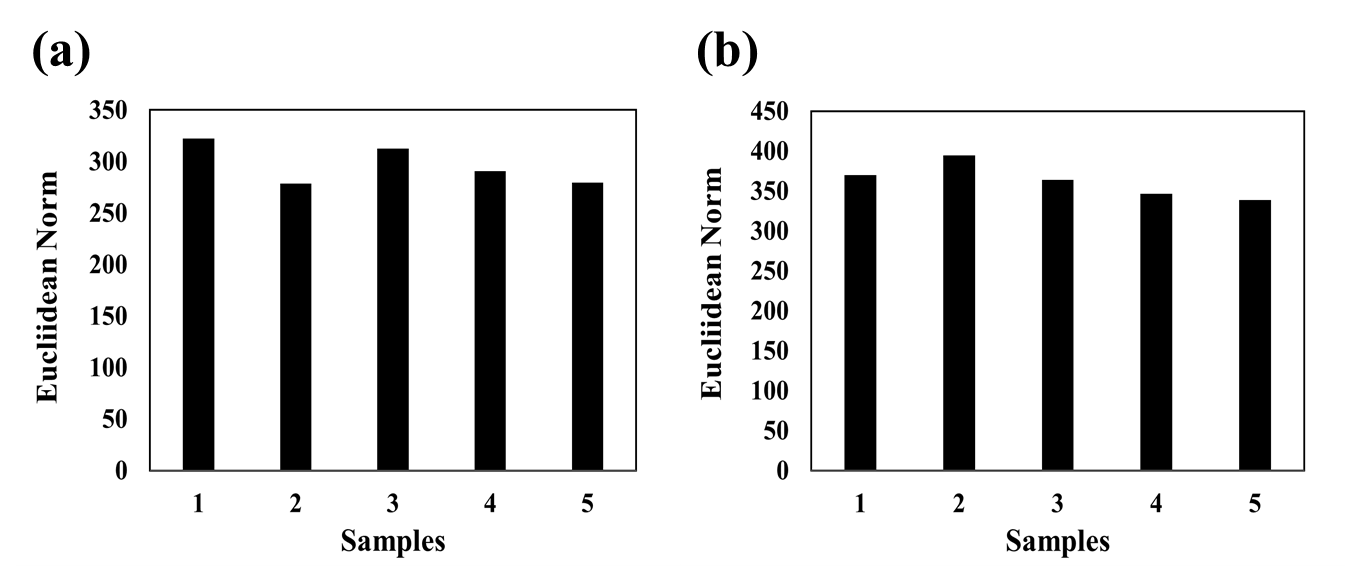


**Fig. S4**. The results of reproducibility of sensor responses after interaction with (a) Non-diabetic and (b) diabetic salivary metabolites. The sensor was fabricated based on optimized condition and its response was captured after 2 min.


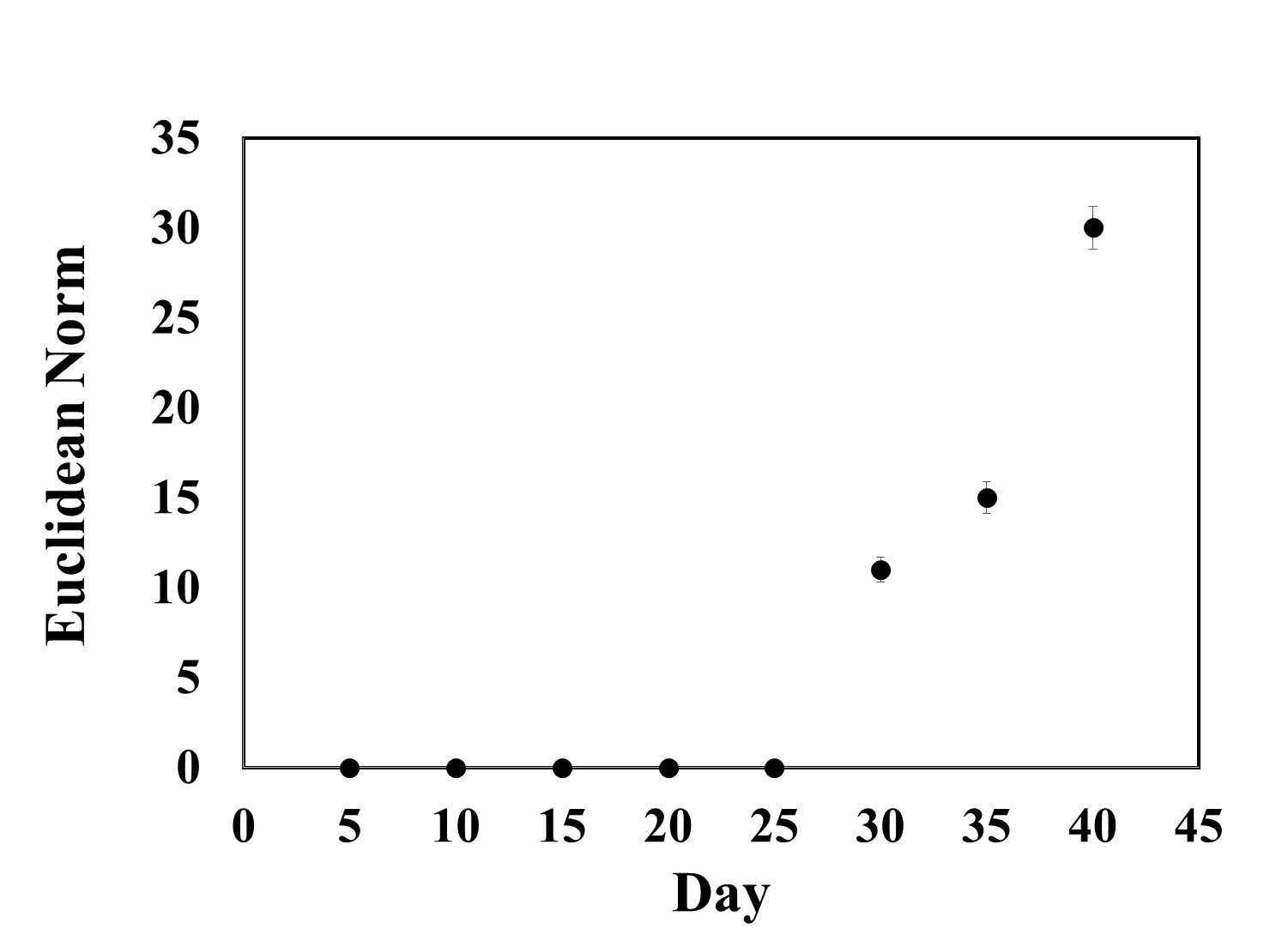


**Fig. S5**. Evaluation of the sensor stability. The sensor was fabricated based on optimized condition.


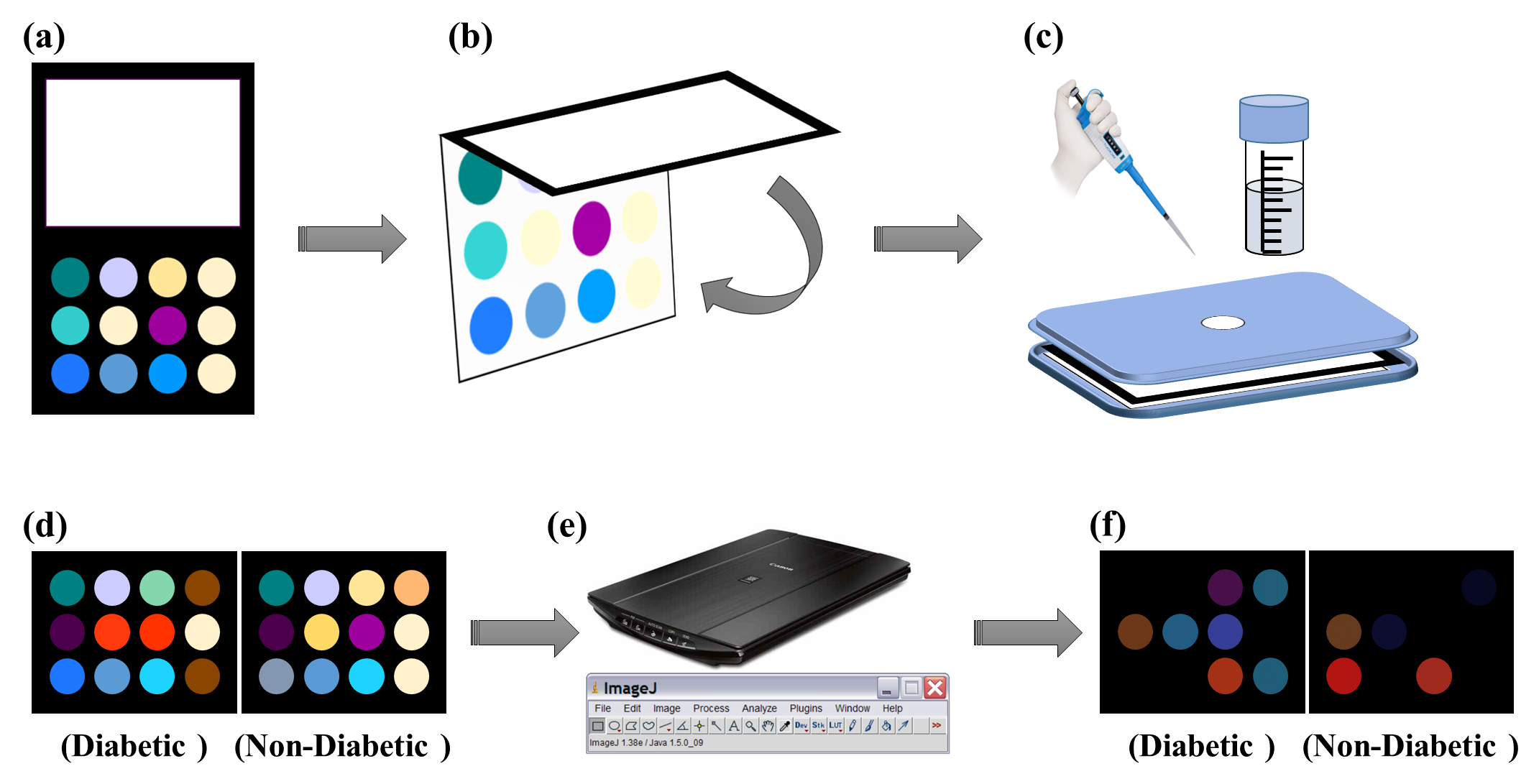


**Figure S6**. The proposed procedure for analysis of salivary metabolite: (a) Fabrication of the sensor, (b) folding the sensor, (c) pressing the sensor between the holders, (d) displaying the sensor responses, (e) capturing and analysis the sensor responses, (f) creating a unique colorimetric pattern for each studied groups.
